# A Wireless Angle and Position Tracking Concept for Live Data Control of Advanced, Semi-Automated Manufacturing Processes

**DOI:** 10.3390/s20092589

**Published:** 2020-05-02

**Authors:** Melanie Lipka, David Meinel, Stefan Müller, Erik Sippel, Jörg Franke, Martin Vossiek

**Affiliations:** 1 Institute of Microwaves and Photonics, University of Erlangen-Nürnberg, 91058 Erlangen, Germany; erik.sippel@fau.de (E.S.); martin.vossiek@fau.de (M.V.); 2 ESE, 38122 Braunschweig, Germany; david@meinel.at; 3 DEPRAG SCHULZ GMBH u. CO., 92224 Amberg, Germany; s.mueller@deprag.de; 4 FAPS, University of Erlangen-Nürnberg, 91058 Erlangen, Germany; joerg.franke@faps.uni-erlangen.de

**Keywords:** industrial automation, worker information system, localization, FMCW, angle-of-arrival

## Abstract

Despite recent industrial automation advances, small series production still requires a considerable amount of manual work, and training, and monitoring of workers is consuming a significant amount of time and manpower. Adopting live monitoring of the stages in manual production, along with the comprehensive representation of production steps, may help resolve this problem. For ergonomic live support, the overall system presented in this paper combines localization, torque control, and a rotation counter in a novel approach to monitor of semi-automated manufacturing processes. A major challenge in this context is tracking, especially hand-guided tools, without the disruptions and restrictions necessary with rigid position encoders. In this paper, a promising measurement concept involving wireless wave-based sensors for close-range position tracking in industrial surroundings is proposed. By using simple beacons, the major share of processing is transferred to fixed nodes, allowing for reduced hardware size and power consumption for the wireless mobile units. This requires designated localization approaches relying on only relative phase information, similar to the proposed Kalman-filter-based-beam-tracking approach. Measurement results show a beam-tracking accuracy of about 0.58${}^{\circ }$ in azimuth and 0.89${}^{\circ }$ in elevation, resulting in an overall tracking accuracy of about 3.18 cm.

## 1. Introduction

**Figure 1:**
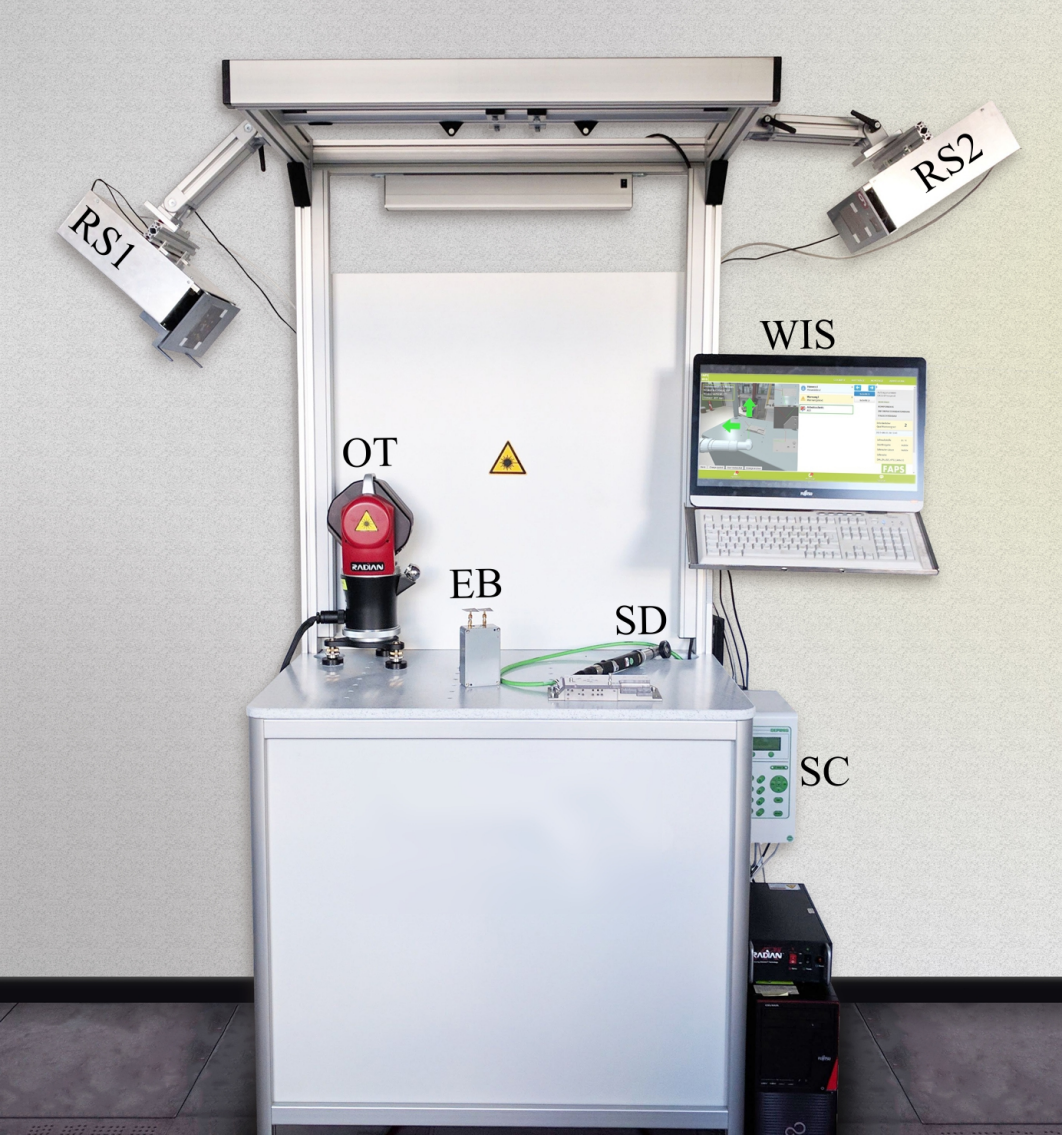
Experimental workstation setup with a worker information system (WIS), an optical tracker (OT) as a reference, a localization system consisting of two 24 GHz remote stations (RS1, RS2), and one mobile experimental beacon (EB), screwdriver (SD), and screwdriver control unit (SC).

The increasing demand for individualization and variant richness creates new challenges for the automation of industrial production. Especially in small batch production, humans may outperform robots, since the cognitive abilities of the former allow for flexible customization and better adaptability. Nevertheless, the partial automation of tools and workspaces, such as the one shown in Figure 1, hand in hand with the simultaneous supply of production relevant information can relief the worker significantly. Therefore, assistance systems have become increasingly popular [1].

One approach arising originally from pure information allocation are worker information systems (WIS) [2,3]. These systems can enable re-configurable assembly lines [4], automatically pass relevant process data to the worker [5], and be used as educational devices to reduce training times [6]. Dynamic information provision, for example, through the use of animation and video, promises even further improvement in comparison to static instructions [7]. To make the future workplace even more comfortable and intuitive, live data are incorporated into the WIS, such as through the use of augmented reality [8,9].

However, live data can provide even more than just instructions. If positional information about the workpiece and tool are available in a WIS, the production process can be supervised and controlled in real time [10,11]. An optimally performing system, especially for small parts, depends heavily on a reliable localization system.

A common solution for tasks involving working stands for manual production is the use of rigid position control stands or gantries [12]. Position detection is based on sensing material measures attached to guide rails, which are usually optical or magnetic incremental scales. With such position control systems, resolution of screwdriving positions with a minimum distance of 5 mm is possible. The main limitations are the working range, which depends on the length of the horizontal arm and guide rails as well as the integration of the screwdriver in a rigid vertical orientation, resulting in the necessity of equally orientated screwdriving positions. Flexible assembly at manual work stations and fastenings using handheld, cordless screwdrivers demands for more flexible positioning solutions.

Therefore, wave-based remote systems are desirable for position control in work stations, as they provide more mobility than gantries [12] or string encoders [13]. A potential solution are visual sensor systems, which were demonstrated in spacious working areas in [14]. These systems come along with only one node [15] but are more robust and provide better coverage by employing multiple sensors [16]. Such approaches work well in the planning phase but may be hampered by dust and steam in harsh environments. Furthermore, privacy concerns can lead workers to oppose camera-based sensor networks. Another approach are ultrasound localization systems, which are often used for indoor close-range applications [17,18], but are affected by ultrasonic noise created by machines or handling processes. Similar systems for manual assembly have been researched [19] and are commercially available [20]. However, feeding technologies and screwdrivers relying on compressed air are still popular and act as ultrasound noise sources.

In contrast, microwave localization systems promise robustness against noise sources common in industrial environments. The capabilities of such microwave-based system in industrial applications have already been demonstrated in large-space scenarios, such as material flow monitoring or vehicle and tool tracking in factory buildings [21,22].

In this paper, an approach optimized for small-space semi-automated work-stands is presented. To improve and assist manual assembly processes, a wireless localization system, WIS, and screwdriver control are combined, enabling live assistance in manual manufacturing processes. In contrast to existing solutions, combinations with the microwave-based localization system yields high freedom of movement and robustness against common industrial ultrasound noise sources. In the following sections, an overview of the entire system is provided. Next, the localization principle and beam-tracker concept is explained. Finally, measurement results for beam-tracking and overall localization are presented, followed by a fail analysis addressing potential full and partial coverage of remote units during the manufacturing process. The accuracy of the approach thereby profits from to the limited distance between beacon and receiver, due to the application. Furthermore, the proposed approach offers a search-space independent computation time as well as advantageous smoothing of erroneous measurements due to the recursive implementation. However, this approach could result in track loss and requires a sufficient measurement rate.

## 2. System Overview

**Figure 2:**
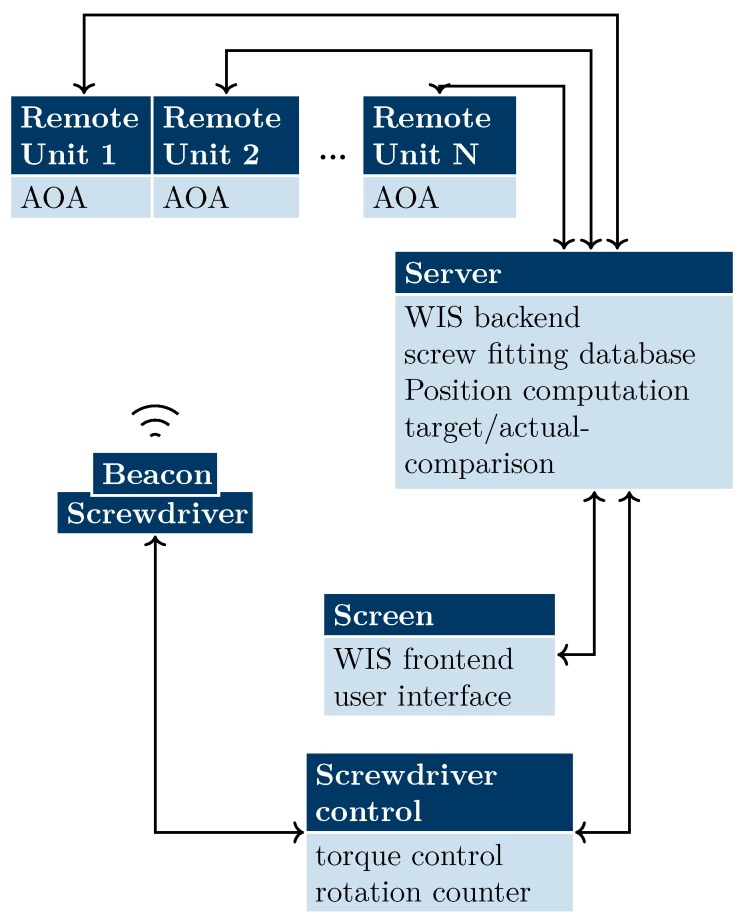
Block diagram of the system components, their tasks, and the communication connections.

The overall system is illustrated in Figure 2. The central element is a server supervising the whole system and merging information from all available sensors and the database. User interactions are received via a graphical user interface (GUI) accessible via touchscreen. The tool is attached to the system via a screwdriver control, which provides the power supply as well as data lines. For localization a beacon is attached to the screwdriver, and multiple remote units are affixed to the working stand. The received signals are processed in the remote units, and the angle results are forwarded to the server to determine a 3D position. A demonstration setup, including a work stand, WIS, screwdriver with control, and a wireless localization system, is shown in Figure 1.

### 2.1. Worker Information and Screwdriver Control

The robust sensor databases provided by modern manual tools make them highly useful in industrial applications. Moreover, when computer-aided-design (CAD) data can be transformed into computer-aided-manufacturing (CAM) data based on algorithms, as in this example, they can be used with sensor data to implement augmented reality applications. In the fastening process, the most important parameters are torque and the angle of rotation. In the system used, the angle is determined directly via the magneto-resistive encoder of the motor and the torque indirectly based on the current of the motor. A sequence controller provides the power supply and the control of the screwdriving sequence. It also stores settings and the setup of fastening strategies defined using a web-based user interface. Additionally, the I/O and fieldbus interface enable communication with higher-order controllers. In the setup described, the sequence controller mainly provides information, such as the status (OK, NOK) and final values of the last fastening. A higher-order controller sets the program number corresponding to the fastening position and unlocks the tool if the screwdriver is localized at the correct position.

**Figure 3:**
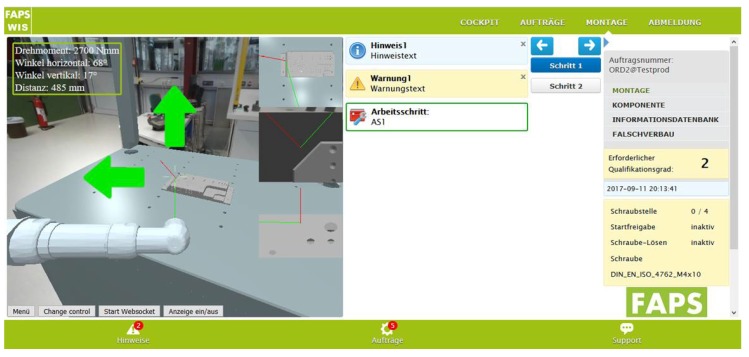
User Interface of the WIS with augmented reality visualization (**left**) and instructions (**right**).

During operation, the tool’s sensor data (torque, velocity, and angle) are processed by a real-time node.JS server that is also permanently receiving and processing the tool position provided by the localization system. Furthermore the WIS has access to stored CAM data, such as positions of screw holes and the geometry of the work-piece. This enables augmented reality with the live positioning of a screwdriver 3D model in a static environment containing the work-piece, workplace, and a 3D image of the surroundings, as shown in Figure 3.

CAM data further enable visualized work steps and support by displaying guide marks, such as directional arrows. The high degree of process data integration and real-time visualization also enables fully automated just-in-time training of new jobs in manufacturing. Thus, a completely flexible allocation of jobs is possible that meets Industry 4.0 requirements for high product individuality. Additionally, mistakes in the screwing process can be detected, and automatic repetitions or rejects can be initiated by the system.

### 2.2. Wireless Localization System

Smooth visualization and reliable adjustment of the screwdriver is only possible if precise permanent tracking in the very limited space of manual work-stations is performed. However, the deployed system must not impair the worker while maintaining the full mobility of the tracked tool. This is accomplished by a microwave-based localization system with remote stations fixed above and, therefore, outside the working area. The mobile transponder mounted at the tool is kept small and lightweight to prevent any additional strain and maintain freedom of movement. Consequently, the main intelligence is transferred to the remote stations and simple incoherent beacons are deployed.

A partial resolution of the multipath and an increase in the signal-to-noise-ratio in the relatively small workspace is achieved by utilizing linear frequency modulated signals. Hence, coarse synchronization by triggering the beacon is necessary to guarantee that the demodulated signal is received within the baseband. Nevertheless, due to partial asynchrony, propagation-time-based approaches, such as time-of-arrival or time-difference-of-arrival, are not possible. Furthermore, the dilution-of-precision (DOP) for multilateration is very poor because the installation conditions require placing all the remote stations close to each other, above the worker, and facing downward.

However, a setup with limited space and distances between beacon and remote stations is favorable for multiangulation, as the absolute deviation depends on angle deviation, as well as the distance to the transponder. Therefore, in this paper, spatially distributed antennas are used to determine the position based solely on angle-of-arrival (AOA). To facilitate this, planar sparse arrays [23] are deployed, and the receiving channels are down-mixed coherently. Calibration [24] removes all unwanted phase shifts due to hardware imperfections, such as receive channel length mismatches. The fixed stations then calculate the arrival angle for the signal, and all stations forward the information to the server for the final position calculation while a new measurement cycle is initiated.

## 3. Localization Principle

To calculate the AOA, the propagation time’s dependence of the signal phase is exploited. When a position measurement is initiated, the beacon emits a linear frequency-modulated signal, which is received at the remote station after a time delay, down-converted with the local ramp of the remote station, and low-pass filtered. The baseband signal [25]

(1) $$\begin{array}{c} S_{\mathrm{BB}}=A_{\mathrm{BB}}\cos\left(2\pi \mu \left(\tau_{m}+\tau_{i}\right)t+\omega_{0}\left(\tau_{m}+\tau_{i}\right)+\phi_{m}\right) \end{array}$$

results, with $A_{\mathrm{BB}}$ denoting the amplitude of the baseband signal, μ the chirp rate, $\tau_{m}$ the unknown time offset due to coarse synchronization, $\tau_{i}$ the time-of-flight to antenna *i* of the sparse array with a total of *I* elements, $\omega_{0}$ the carrier frequency, and $\phi_{m}$ the characteristic unknown phase term for remote unit *m*. The corresponding phase of the down-converted signal,

(2) $$\begin{array}{c} \varphi_{i}=\omega_{0}\left(\tau_{m}+\tau_{i}\right)+\phi_{m}, \end{array}$$

is range-dependent but cannot be evaluated directly due to the unknown time offset and phase term. As $\tau_{m}$ and $\phi_{m}$ are the same for all the coherent receiving channels *i* of one remote station *m*, they can be removed by taking the difference between two phase values,

(3) $$\begin{array}{c} \Delta \varphi_{ij}=\varphi_{i}-\varphi_{j}=\omega_{0}\left(\tau_{i}-\tau_{j}\right)=\omega_{0}\frac{r_{i}-r_{j}}{c_{0}}, \end{array}$$

where $r_{i},r_{j}$ denotes the distance from the beacon to the antenna element *i* and *j*, and $c_{0}$ is the speed of light. Incident angles cause a characteristic phase difference pattern due to the slightly different path lengths from the beacon to the spatially distributed antennas. To determine the angle of arrival, conventional delay-and-sum (DAS) beamformer, Capon beamformer, or subspace-based methods [26] are commonly deployed. In the following section, an approach based on an extended Kalman filter (EKF) is presented for tracking the angle of arrival in individual, fixed stations without sampling the angle-search-space.

### 3.1. State Vector

**Figure 4:**
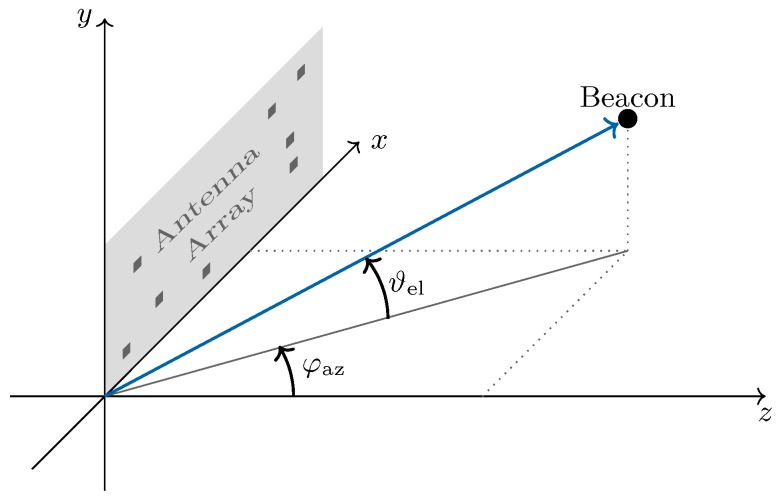
Definition of the azimuth and elevation angles in the antenna array coordinate system.

First, the state vector $x_{k-1|k-1}$ of the beam-tracking problem at the time (k−1) is defined as

(4) $$\begin{array}{c} x_{k-1|k-1}=\left(\begin{array}{c} \varphi_{az}\\ \vartheta_{el}\\ \omega_{\varphi_{az}}\\ \omega_{\vartheta_{el}} \end{array}\right) \end{array}$$

with azimuth angle $\varphi_{az}$ and elevation angle $\vartheta_{el}$ in the array coordinate system (see Figure 4) and the corresponding angular speeds, $\omega_{\varphi_{az}}$ and $\omega_{\vartheta_{el}}$, which enable a rough prediction. The next state is calculated in two steps, the prediction and the update [27,28], as described in the next sections.

### 3.2. EKF Predict

To calculate a prognosis of the likely new position based on the data of the last state, an equation of motion is applied. Considering a movement with constant angle velocity and comparable with the constant velocity approach [29], the occurring accelerations are incorporated into the process noise covariance matrix *Q*. This results in the linear state transition

(5) $$\begin{array}{cc} \; & x_{k|k-1}=F\;\cdot \;x_{k-1|k-1}=\left(\begin{array}{cccc} 1 & 0 & \Delta T & 0\\ 0 & 1 & 0 & \Delta T\\ 0 & 0 & 1 & 0\\ 0 & 0 & 0 & 1 \end{array}\right)x_{k-1|k-1}, \end{array}$$

with *F* denoting the state transition matrix and ΔT the time between two sampling points, and the noise covariance matrix

(6) $$\begin{array}{c} Q=\sigma_{a}^{2}\left(\begin{array}{cccc} \frac{\Delta T^{4}}{4} & 0 & \frac{\Delta T^{3}}{2} & 0\\ 0 & \frac{\Delta T^{4}}{4} & 0 & \frac{\Delta T^{3}}{2}\\ \frac{\Delta T^{3}}{2} & 0 & \Delta T^{2} & 0\\ 0 & \frac{\Delta T^{3}}{2} & 0 & \Delta T^{2} \end{array}\right) \end{array}$$

which is dependent on the variance of the neglected accelerations, $\sigma_{a}^{2}$. For uniform movements, this variance can be set rather low, while rapid turnarounds can only be tracked if the variance is high enough. Along with the measurement noise covariance, the choice of the process noise covariance provides a way to balance the Kalman filter and determine how much trust the filter puts into predicting and updating. Large *Q* values increase trust in the measurement, while large values in *R* put more weight on the prediction.

So far, no measurement data have been incorporated, so the uncertainty of the state rises, which is reflected in the larger values of the predicted state covariance matrix,

(7) $$\begin{array}{cc} P_{k|k-1} & =F\;\cdot \;P_{k-1|k-1}\;\cdot \;F^{T}+Q, \end{array}$$

where $P_{k-1|k-1}$ denotes the noise covariance matrix of the last state at time k−1.

### 3.3. EKF Update

Next, the measurement data are used to update the predicted state. The measurement vector

(8) $$\begin{array}{cc} z_{k} & =\left(\begin{array}{c} \Delta \varphi_{12}\\ \vdots \\ \Delta \varphi_{ij}\\ \vdots \\ \Delta \varphi_{I(I-1)} \end{array}\right)=h\left(x_{k}\right)+w_{k} \end{array}$$

is related to the state vector $x_{k}$ via an output transition function vector $h_{k}$ with added Gaussian noise $w_{k}$. This relationship between the azimuth and elevation angles (state) and the phase difference (measurement) can be expressed as

(9) $$\begin{array}{cc} \; & h_{ij}=\varphi_{i}-\varphi_{j}=\\ \; & =\frac{2\pi }{\lambda_{0}}\left[\left(x_{i}-x_{j}\right)\sin\left(\varphi_{az}\right)\cos\left(\vartheta_{el}\right)+\left(y_{i}-y_{j}\right)\sin\left(\vartheta_{el}\right)\right], \end{array}$$

with $h_{ij}$ denoting the transition function vector element for antenna elements *i* and *j*, $x_{i}$; $y_{i}$; $x_{j}$ and $y_{j}$ the 2D coordinates of the antenna elements *i* and *j* within the used planar sparse array, and $\lambda_{0}$ the wavelength at $\omega_{0}$.

To merge the predicted state $x_{k|k-1}$ and the measurements $z_{k}$, the Kalman gain,

(10) $$\begin{array}{cc} K_{k} & =P_{k|k-1}H_{k}^{T}{\left(H_{k}P_{k|k-1}H_{k}^{T}+R\right)}^{-1}, \end{array}$$

has to be calculated with *R* denoting the measurement noise covariance matrix and the output transition matrix $H_{k}$, which is obtained by forming the Jacobian matrix

(11) $$\begin{array}{c} H_{k}=\left(\begin{array}{cccc} \frac{\partial h_{12}}{\partial \varphi_{az}} & \frac{\partial h_{12}}{\partial \varphi_{el}} & 0 & 0\\ \vdots & \vdots & \vdots & \vdots \\ \frac{\partial h_{ij}}{\partial \varphi_{az}} & \frac{\partial h_{ij}}{\partial \varphi_{el}} & 0 & 0\\ \vdots & \vdots & \vdots & \vdots \\ \frac{\partial h_{I(I-1)}}{\partial \varphi_{az}} & \frac{\partial h_{I(I-1)}}{\partial \varphi_{el}} & 0 & 0 \end{array}\right) \end{array}$$

of $h(x_{k|k-1})$. As the output transition function does not depend on angular velocity, the derivation and, therefore, the right columns are zero. For the left coloums,

(12) $$\begin{array}{cc} \; & \frac{\partial h_{ij}}{\partial \varphi_{az}}=\frac{2\pi }{\lambda_{0}}\left[\left(x_{i}-x_{j}\right)\cos\left(\vartheta_{el}\right)\;\cdot \;\cos\left(\varphi_{az}\right)\right], \end{array}$$

(13) $$\begin{array}{cc} \; & \frac{\partial h_{ij}}{\partial \vartheta_{el}}=\frac{2\pi }{\lambda_{0}}\left[\left(x_{j}-x_{i}\right)\sin\left(\varphi_{az}\right)\sin\left(\vartheta_{el}\right)+\left(y_{i}-y_{j}\right)\cos\left(\vartheta_{el}\right)\right] \end{array}$$

is obtained. For the measurement noise covariance matrix, the individual measured phases are considered uncorrelated and Gaussian distributed. Noise in FMCW radars originates from manifold sources [30], either resulting directly in phase noise, for example by phase-locked-loop or oscillator noise, or considered as additive noise, such as background noise or man-made interference signals. Therefore, the overall noise is a sum of various random processes, and the central limit theorem [31] holds. For additive white Gaussian noise, it should be noted that it induces Gaussian distributed phase noise [32] for sufficient signal-to-noise-ratios, which is common in close-range applications. Since the measurement vector in Equation (8) contains all possible phase differences, each phase is evaluated several times. Therefore, the measurement noise covariance matrix is non-diagonal, and four different entry values depending on the phase variance $\sigma_{p}^{2}$ arise:

- Uncorrelated differences with no phase value in common:
  (14) $$\begin{array}{c} \mathrm{Cov}(\varphi_{i}-\varphi_{j},\varphi_{n}-\varphi_{m})=0,\\ \mathrm{with}\;i\neq j\neq n\neq m\in [0,...,I]. \end{array}$$
- Main diagonal elements:
  (15) $$\begin{array}{cc} \mathrm{Var}(\varphi_{i}-\varphi_{j}) & =\mathrm{Var}\left(\varphi_{i}\right)+\mathrm{Var}\left(\varphi_{j}\right)=2\sigma_{p}^{2}. \end{array}$$
- Differences with one phase value $\varphi_{i}$ in common:
  (16) $$\begin{array}{cc} \; & \mathrm{Cov}\left(\varphi_{i}-\varphi_{j},\varphi_{i}-\varphi_{m}\right)=\sigma_{p}^{2}. \end{array}$$
- Differences with one phase value $\varphi_{i}$ in common but with a different sign:
  (17) $$\begin{array}{cc} \; & \mathrm{Cov}\left(\varphi_{i}-\varphi_{j},-\varphi_{i}+\varphi_{m}\right)=-\sigma_{p}^{2}. \end{array}$$

Using the Kalman gain, the predicted state $x_{k|k-1}$ and covariance matrix $P_{k|k-1}$ is now updated to calculate the new state and corresponding covariance [27,28],

(18) $$\begin{array}{cc} x_{k|k} & =x_{k|k-1}+K_{k}\left(z_{k}-h\left(x_{k|k-1}\right)\right), \end{array}$$

(19) $$\begin{array}{cc} P_{k|k} & =\left(I_{4}-K_{k}H_{k}\right)P_{k|k-1}, \end{array}$$

with the 4×4 identity matrix $I_{4}$. $P_{k|k}$ and $x_{k|k}$ denote the final state for this Kalman filter pass, which is also the starting point for the next cycle.

## 4. Measurement Setup and Results

For verification, a localization measurement setup consisting of three remote units, one beacon, and a high precision reference provided by a Stäubli TX90-XL robotic arm with a repetition accuracy of ±0.03 mm was used. To account for typical work-stand conditions, a measurement area of 1 m × 1 m × 0.3 m was chosen, and the remote units are installed above the measurement area. The remote units and the beacon used the frequency-modulated-continuous-wave (FMCW) principle [25] with a bandwidth of 250 MHz at a carrier frequency of 24 GHz (industrial-scientific-medical band), a ramp duration of 1024 μs, and a transmit power of 13 dBmW. As the application scenario requires only one tool to be localized, only one beacon was used. Coarse synchronization was carried out by triggering the beacon via a frequency-shift-keying sequence, which then started emitting a linear frequency modulated signal. A computationally-expensive fine synchronization [33,34] was omitted, as it would have required a sophisticated computation module in the mobile beacon.

**Figure 5:**
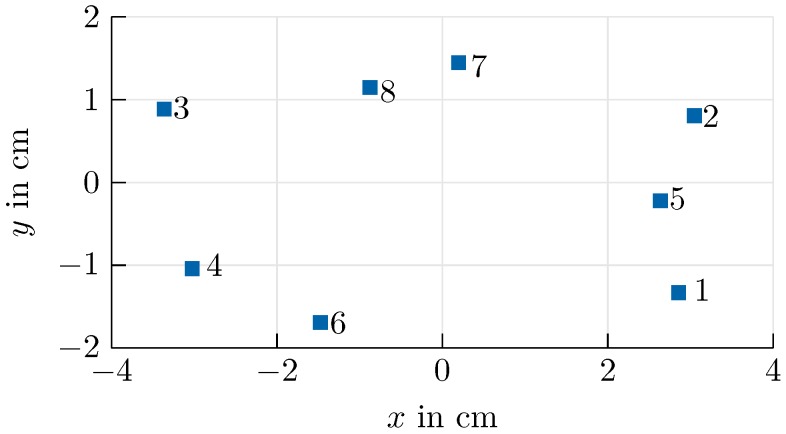
Antenna positions in the sparse array used for angle determination.

At each remote station, a planar sparse array [23] consisting of eight receiving elements arranged as shown in Figure 5 was deployed. Coherency among the channels was established by down-converting all channels of one remote station with the same local ramp. The phase mismatch caused, for example, by slightly different path lengths in the experimental system, as well as the positions of the remote stations, was determined by calibration [24] prior to of the measurements.

Base-band signals were low-pass filtered with a cutoff frequency of 5 GHz and fast-Fourier-transformed using the internal Xilinx Zynq Module at the remote stations. The range dependent phase (Equation (2)) for all eight channels was evaluated at the maximum of the sum of magnitudes of the baseband spectra. For the following results, the proposed EKF was applied to calculate the AOA for the coherent channels at the individual stations. In parallel, a classical delay-and-sum (DAS) beamformer using the same dataset was implemented for comparison. Subsequently, the angle calculation results were exploited for 3D localization involving multiple fixed, remote stations. Finally, susceptibility to failure is analyzed by discussing potential worst-case scenarios.

### 4.1. Beam-Tracking Performance

**Figure 6:**
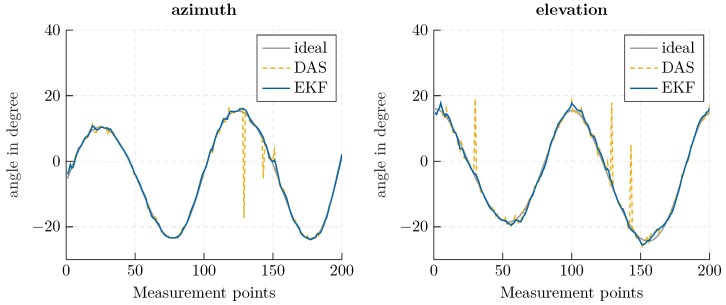
Azimuth (**left**) and elevation (**right**) angle for a classical delay-and-sum (DAS) beamformer (yellow, dashed) and EKF-based approach (blue, solid), in comparison to the ideal angle (gray, solid).

**Figure 7:**
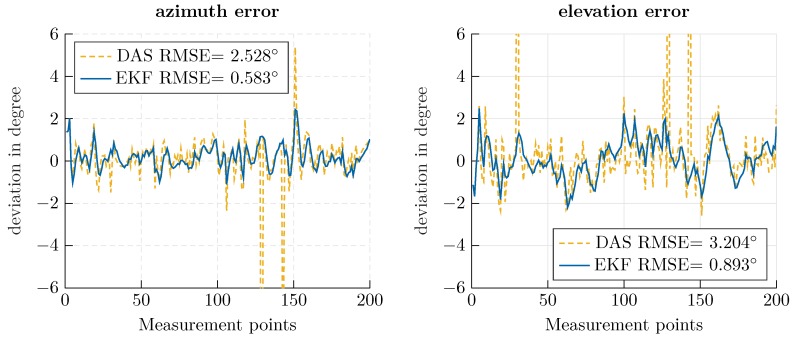
Deviation from the ideal angle for azimuth (**left**) and elevation (**right**) for the delay-and-sum (DAS) beamformer (yellow, dashed) and EKF-based approach (blue, solid).

Using the beam-tracking algorithm from Section 3, an exemplary helix-shaped trajectory with 200 measurement points was evaluated. The Kalman filter’s initial state $x_{k|k}$ was determined using a conventional DAS beamformer and presuming a standard deviation of 30${}^{\circ }$ to derive the covariance matrix $P_{k|k}$. In Figure 6, a direct comparison of the EKF and a conventional beamformer is shown. The proposed approach offers increased accuracy, especially for outlying measurements; since the previous state is considered in the prediction calculation, outliers are smoothed out. In Figure 7, the deviation from the robotic reference is illustrated, and the corresponding RMSE is indicated. The root mean square error was calculated as

(20) $$\begin{array}{c} RMSE_{\mathrm{az}/\mathrm{el}}=\sqrt{\frac{\sum_{k=1}^{K}{\left(\varphi_{\mathrm{meas},k}-\varphi_{\mathrm{ideal},k}\right)}^{2}}{K}}, \end{array}$$

with $\varphi_{\mathrm{meas},k}$ denoting the calculated angles, $\varphi_{ideal,k}$ the ideal angle values for azimuth and elevation at measurement point *k*, and *K* the number of data points.

Using the proposed approach, an RMSE of less than one degree could be achieved. By comparing the RMSE for azimuth and elevation, the impact of the aperture size is made visible. A greater accuracy is achieved for the azimuth angle, which corresponds to the array expansion in the *x*-direction in Figure 5. Furthermore, the smoothing properties of the Kalman filter can be seen in the mitigation of outlying measurements.

Concerning the computational effort required by the approaches, independent of the angle search space, the most complex operation in the Kalman filter is the inversion of an $\frac{I(I-1)}{2}\times \frac{I(I-1)}{2}$ matrix. In contrast, the computational effort for a DAS beamformer scales with the angle range and refinement of the search space, which is necessary for sufficient accuracy. For the series of measurements shown in Figure 6 and Figure 7, the beamformer sampled an angle value range of [−30${}^{\circ }$, 30${}^{\circ }$] with a resolution of 0.6${}^{\circ }$, which implies the calculation and matching of 10,000 hypothetical phases. The EKF for an eight element antenna array, in comparison, required the inversion of an 28×28 matrix. Therefore, the EKF approach is especially appropriate when a fine angle resolution or large search space is necessary.

### 4.2. 3D Tracking Results

**Figure 8:**
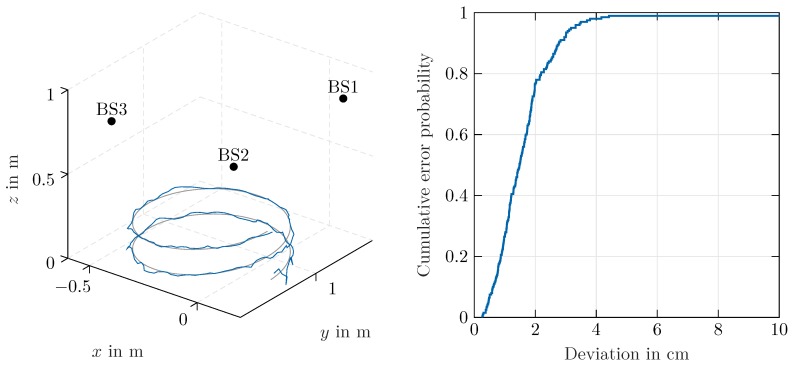
3D view of the ideal (gray) and measured (blue) trajectories and cumulative error probability function.

After the angle calculations are performed for three stations, the results were forwarded to the server for 3D localization. As only bearing information was available, the position was calculated based on multiangulation [35,36]. In this paper, the bearings-only approach presented in [37] was applied. A 3D view of the ideal and measured helix trajectory and the resulting cumulative error probability density function are shown in Figure 7 and Figure 8. The resulting RMSE error, in accordance with [38], was calculated as

(21) $$\begin{array}{c} RMSE_{k}=\sqrt{\frac{\sum_{K}^{k=1}\left(\Delta x_{k}^{2}+\Delta y_{k}^{2}+\Delta z_{k}^{2}\right)}{K}}, \end{array}$$

with $\Delta x_{k}$, $\Delta y_{k}$, $\Delta z_{k}$ denoting the difference between the measured and ideal positions in Cartesian coordinates. For the trajectory in Figure 8, located at a distance of about 1 m from the remote stations, an overall RMSE of 3.18 cm was achieved. The accuracy strongly profits from the relatively small distance to the remote stations. Although angle determination is possible as long as a line-of-sight with sufficient signal-to-noise ratio exists, for beacons located further apart, a stronger impact of the angle error on the overall localization accuracy can be expected.

### 4.3. Fail Analysis

The localization system is also involved in safety critical tasks, such as position triggered displaying of information and warnings for accident prevention. Therefore, it is important to provide the position even if individual antennas or stations are covered or malfunction.

Concerning the beam-tracker, a minimum of three antenna elements is necessary to prevent a complete failure. Thereby, accuracy is proportional to the aperture, therefore the closest spaced antenna elements serve as the worst case in the following section.

**Figure 9:**
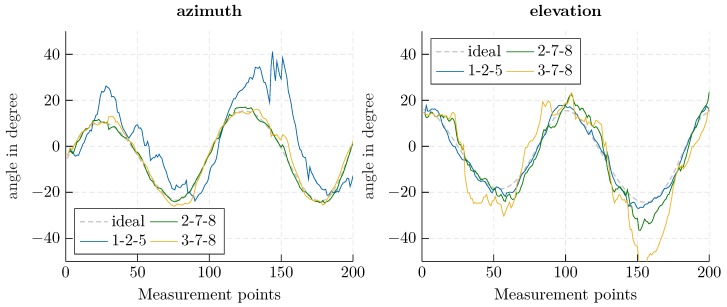
Beam-tracking results for the three worst-case scenarios involving only three of eight antenna elements with a small aperture.

Considering the sparse array used in the experimental setup, as illustrated in Figure 5, the smallest apertures are achieved with the antenna combinations 3-7-8, 2-7-8, and 1-2-5, while the largest is achieved with the combination 2-3-6. As shown in Figure 9, beam-tracking accuracy is reduced significantly for the unfavourable worst-case coverages. The RMSEs (20), presented in Table 1, indicate that it is not possible to provide reliable angle information in this case.

For comparison, the performance of the three antenna combination with the largest aperture and the full array’s performance are listed in Table 1. Since accuracy is mainly defined by the outermost elements, the malfunction of an inset antenna reduces it only slightly in comparison to the full array’s performance. In conclusion, the inner antennas and, therefore, the number of channels may be reduced, while the beam-tracking approach still yields similar results due to the recursive implementation. Thus, the computational complexity of the matrix inversion is reduced, but a higher sensitivity to erroneous phase values arises since less measurement information is available. Consequently, the uniqueness range is reduced, so the measurement rate has to be sufficiently high to keep track of the angle. Therefore, for fast movements, the Kalman filter may lose track and has to be initialized again.

Equally, if the number of channels is maintained, the inter-element spacing may be increased, resulting in higher accuracy due to the larger aperture. Hence, a trade-off between the measurement rate and array size has to be found to achieve the best accuracy without losing the track.

The second fail case is the outage of a complete station. This may be caused by a hardware failure or an intended shutoff if the beam-tracker fails due to unfavorable coverage. A point of intersection for multiangulation can only be calculated if at least two functioning stations remain in the setup. However, the reduced number of stations implies less redundancy in the system and a reduced aperture of all remote stations.

**Figure 10:**
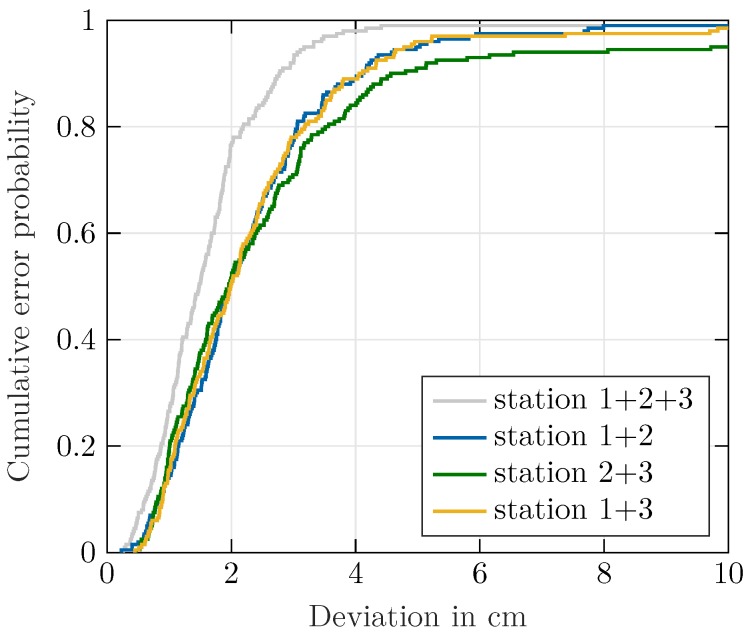
Overall localization results for the worst case of one station failing for a whole measurement cycle.

Figure 10 shows the cumulative error probability for a station failure over the whole measurement cycle. It can be seen that localization accuracy degrades due to missing information, but localization with reduced accuracy is possible. Nevertheless, the results in Figure 10 show that the failure of Station 1 would have the biggest impact. This is mainly caused by outliers at the beginning of the measurement cycle, which cannot be effectively smoothed due to missing information from one station. Especially in the settling time, the Kalman filter is more vulnerable to severe outliers and overshooting of the filter output due to the high state covariance.

## 5. Conclusions

In this paper, a novel approach for live-data-driven support in semi-automated manufacturing processes was presented. Tracking and monitoring, in collaboration with automatic position-dependent information allocation, promises significant improvement in the assembly process. In particular, wireless approaches resolve problems of established systems and enable more flexibility in movement. Angle-based localization concepts offer the opportunity for further simplification of the beacons by relying on phase information only and forgoing computationally complex synchronization procedures and benefit from the small distances in usual manual workstations. The optimized Kalman-filter-based approach for angle determination proposed in this paper offers increased accuracy and is also independent of the search space size. With the proposed beam-tracker, an RMSE error of less than one degree for azimuth and elevation and an overall localization accuracy in the cm range was achieved. The fail analysis shows that beam-tracking accuracy strongly depends on the subset of antennas remaining for localization. Omitting the evaluation of the inner antennas yields almost no accuracy degradation and thus larger element spacing and accuracy improvement is possible. Nevertheless, this comes at the expense of a smaller uniqueness range, which requires higher measurement rates. The recursive implementation also supports localization if a complete stations fails as long as at least two remote stations receive a line-of-sight signal from the beacon.

## Figures and Tables

**Table 1:** RMSEs for combinations of three antenna elements.

Antenna Combination	Aperture	${\mathrm{RMSE}}_{\mathrm{az}}$	${\mathrm{RMSE}}_{\mathrm{el}}$
3-7-8	small elevation expansion	$2.194^{\circ }$	$10.731^{\circ }$
2-7-8	small elevation expansion	$1.051^{\circ }$	$4.764^{\circ }$
1-2-5	small azimuth expansion	$13.227^{\circ }$	$1.964^{\circ }$
2-3-6	outermost elements	$0.668^{\circ }$	$0.974^{\circ }$
full array	all elements	$0.583^{\circ }$	$0.893^{\circ }$
